# Supplementary material for: Towards unified AI-driven fracture mechanics: the extended deep energy method (XDEM)
Source: Nat Commun. 2026 Aug 15;17:8492. doi: 10.1038/s41467-026-76748-1 (PMC13484471; doi:10.1038/s41467-026-76748-1)
Supplement: Supplementary file 3 [file 41467_2026_76748_MOESM3_ESM.zip › pic/Results/Stress_intensity_factor/Mixed/beta_30/crack_detailed_fields.pdf]

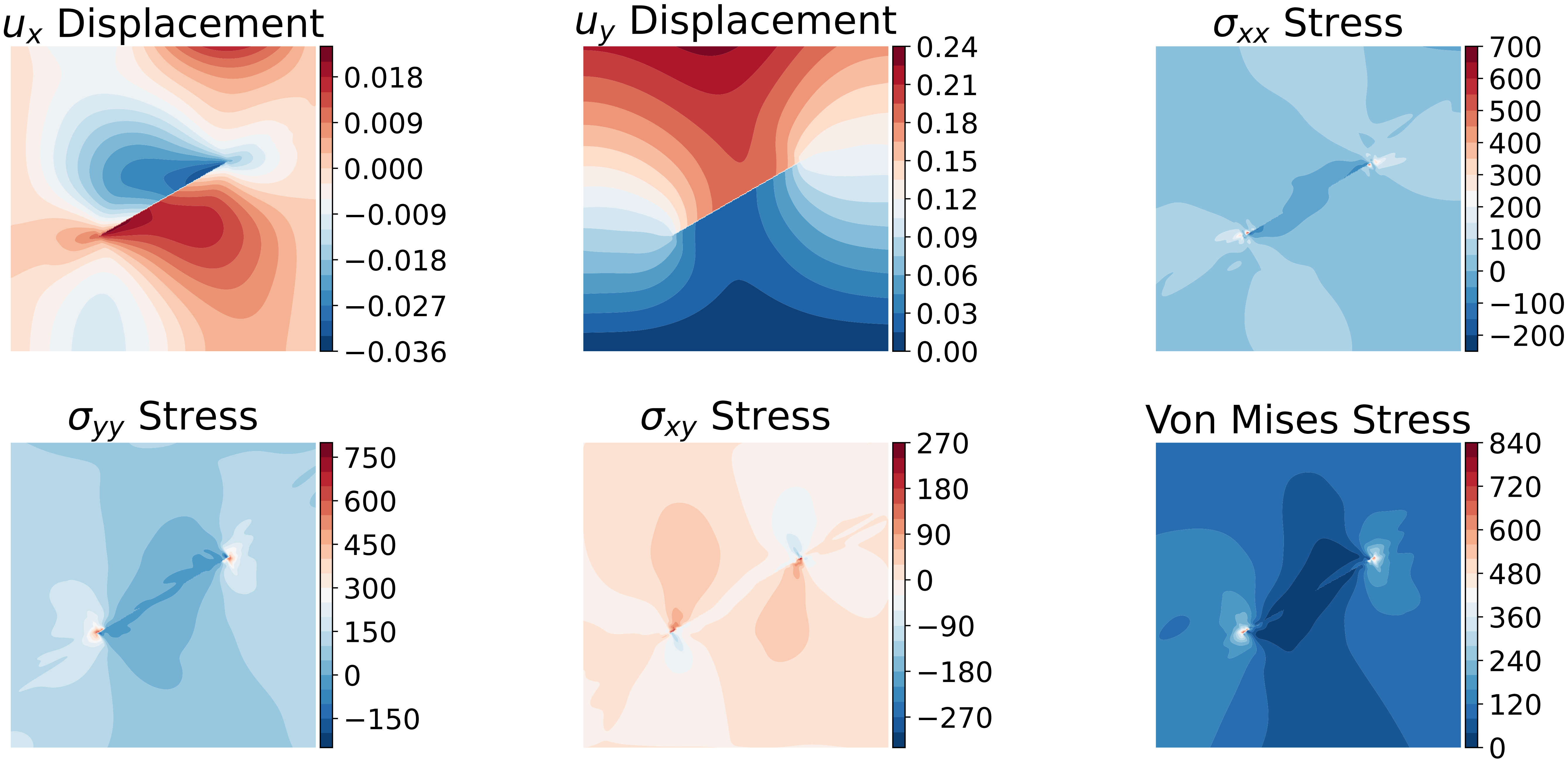

u_x Displacement
0.018
0.009
0.000
−0.009
−0.018
−0.027
−0.036
u_y Displacement
0.24
0.21
0.18
0.15
0.12
0.09
0.06
0.03
0.00
σ_{xx} Stress
700
600
500
400
300
200
100
0
−100
−200
σ_{yy} Stress
750
600
450
300
150
0
−150
σ_{xy} Stress
270
180
90
0
−90
−180
−270
Von Mises Stress
840
720
600
480
360
240
120
0
